# What predicts willingness to engage in consensual non-monogamous relationships? A study among Italian emerging adults

**DOI:** 10.1192/j.eurpsy.2025.2406

**Published:** 2025-08-26

**Authors:** L. Ghidetti, S. Moscatelli, Y. Koc, S. Ciaffoni

**Affiliations:** 1 Psychology, University of Bologna, Bologna, Italy; 2 Behavioural and Social Sciences, University of Groningen, Groningen, Netherlands

## Abstract

**Introduction:**

Consensual non-monogamous relationships (CNM) are characterized by a diversity of approaches to intimate relationships, diverging from the traditional monogamous model and exploring new possibilities. CNM relationships include forms such as polyamory, open relationships, and swinging. There are no official and systematic epidemiological data on CNM relationships in Italy; however, these relationships have recently gained more visibility due to social media, support groups, and open discussions about relationship diversity that are slowly emerging in a country whose culture is traditionally influenced by Catholicism and conservative social norms.

**Objectives:**

Most research focuses on discrimination against individuals involved in CNM, while the factors underlying people’s engagement in such relationships are overlooked. This study aims to investigate predictors of willingness to engage and actual engagement in CNM among Italian emerging adults. Specifically, based on the Theory of Planned Behavior (Ajzen, 1991), we examined the role of perceived social norms and perceived behavioral control, as well as other predictors already tested in previous research.

**Methods:**

Participants completed an online questionnaire examining factors such as gender, sexual orientation, willingness to engage in CNM relationships (WECNM; Moors et al., 2015; 6 items), avoidant and anxious attachment style (ECR-S; Wei et al., 2007; 12 items), erotophilia (ATP; Johnson et al., 2015; 5 items), social norms (PSN; custom scale, 4 items), perceived behavioral control in CNM relationships (PBC; custom scale, 2 items). Data were analyzed using SPSS, applying multiple linear regression.

**Results:**

The sample consists of 667 emerging adults (*M_age_* = 23.29; age range: 18 – 30) with diverse sexual orientations and gender identities. Of these, 535 participants are involved in monogamous relationships, 65 in polyamorous, and 67 in open relationships. In line with our hypotheses, results show that social norms, perceived behavioral control, non-heterosexual orientation, and cisgender male identity are significant predictors of both the willingness to engage in and the commitment to consensual non-monogamous relationships. Avoidant attachment style, as well as erotophilia, predict the predisposition but not the actual commitment to CNM.

**Conclusions:**

The results contribute to a deeper understanding of CNM relationships in the Italian context by identifying some individual and social factors related to openness and involvement in CNM relationships. This provides preliminary evidence of the utility of the Theory of Planned Behavior in understanding this type of relationship. Moreover, the significant number of individuals involved or interested underscores the importance of considering relationship diversity in future research and social policies, with the aim of promoting greater acceptance and inclusion.

**Disclosure of Interest:**

None Declared

